# Three-Dimensional Transesophageal Echocardiography Is Useful for Preventing Prosthetic-Patient Mismatch After Surgical Aortic Valve Replacement

**DOI:** 10.3390/jcm14134762

**Published:** 2025-07-05

**Authors:** Kazuki Yoshida, Haruka Sasaki, Hiroyuki Takaoka, Moe Matsumoto, Yusei Nishikawa, Yoshitada Noguchi, Shuhei Aoki, Katsuya Suzuki, Satomi Yashima, Makiko Kinoshita, Noriko Suzuki-Eguchi, Shuichiro Takanashi, Kazuyuki Matsushita, Goro Matsumiya, Yoshio Kobayashi

**Affiliations:** 1 Department of Laboratory Medicine, Chiba University Hospital, Chiba 260-8677, Japan; 2 Department of Cardiovascular Medicine, Chiba University Graduate School of Medicine, Chiba 260-0856, Japan; 3 Kawasaki Heart Center, Kawasaki Saiwai Hospital, Kanagawa 212-0014, Japan; 4 Department of Cardiovascular Surgery, Chiba University Graduate School of Medicine, Chiba 260-0856, Japan

**Keywords:** surgical aortic valve replacement, prosthesis-patient mismatch, recommended prosthetic aortic valve size, aortic valve annulus area-derived diameter, three-dimensional transesophageal echocardiography

## Abstract

**Introduction**: Prosthesis-patient mismatch (PPM) in surgical aortic valve replacement (SAVR) is known to be a poor prognostic factor. However, the parameters for preventing postoperative PPM in SAVR have not been established. **Materials and Methods**: Two hundred and five patients (mean age 72.5 ± 7.4 years, 129 males) who underwent SAVR were analyzed. Preoperatively, we determined the recommended prosthesis valve size from the mean aortic valve (AV) diameter derived from the AV annulus area by preoperative three-dimensional transesophageal echocardiography (3D-TEE). We investigated the association between pre- and postoperative changes in annulus diameter and the occurrence of PPM. **Results**: PPM was present in 6 patients (2.9%). Pre- and postoperative AV annulus diameter change ratio was greater in the group with PPM than in that without PPM (10.4 ± 3.6% vs. 3.0 ± 5.6%, *p* = 0.002). The use of prosthetic valve rings smaller than the recommended size was higher in the group with PPM than in that without PPM. (83.3% vs. 20.6%, *p* = 0.002). On multivariate logistic regression analysis, use of a valve smaller than the recommended size was an independent predictor of PPM (odds ratio 19.3, 95% confidence interval 2.14–174.5, *p* = 0.008). **Conclusions**: The recommended prosthetic AV size based on preoperative 3D-TEE is useful for determining the optimal prosthetic AV size to prevent PPM after SAVR.

## 1. Introduction

Prosthesis-patient mismatch (PPM) is a condition in which the effective orifice area (EOA) is small relative to body size after surgical aortic valve replacement (SAVR) [1]. EOA index (EOAi), namely the value of EOA corrected for body surface area (BSA), is used as an index of PPM. An EOAi < 0.65 cm^2^/m^2^ is defined as severe PPM, and known to be a poor prognostic factor after SAVR [2,3]. To minimize the risk of PPM, prosthetic aortic valve (AV) size is usually selected based on BSA in the individual patient [4]. However, the size of the native AV annulus does not always correlate with BSA, and PPM can occur even when valve sizing is guided by BSA [5,6]. Therefore, a more precise indicator is needed to prevent PPM.

Three-dimensional transesophageal echocardiography (3D-TEE) is useful in detailed evaluation of measurement of the AV annulus [7]. In our practice, we preoperatively calculate the recommended prosthesis valve size from mean AV diameter derived from the AV annulus. Here, we investigated the usefulness of these indicators in preventing PPM.

## 2. Materials and Methods

We retrospectively studied 210 consecutive patients who underwent SAVR using a bioprosthetic valve replacement for a native valve with preoperative 3D TEE at Chiba University Hospital or Kawasaki Saiwai Hospital between January 2017 and May 2023. We excluded two patients who had not undergone postoperative transthoracic echocardiography (TTE), two patients with a left ventricular outflow tract (LVOT) flow greater than 1 m/s on postoperative TTE, and one patient whose preoperative TEE images were poor and could not be analyzed. Finally, 205 patients were available for analysis. Preoperative TTE was performed in all patients. Before discharge, patients were evaluated for PPM by postoperative TTE. We defined severe PPM as an EOAi of 0.65 cm^2^/m^2^ or lower with postoperative TTE based on previous reports [5,8,9]. We compared patient characteristics, echocardiographic characteristics, and procedural techniques between the groups with and without severe PPM. This study was approved by the institutional review board of Chiba University Graduate School of Medicine (Approval no. HK202408-05).

### 2.1. Transthoracic Echocardiography

TTE was performed pre- and postoperatively as routine clinical practice using the EPIQ system with an X5-1 transducer; the iE33 system with an S5-1 transducer (Philips Medical Systems, Andover, MA, USA); or the Vivid E9 system with an M5S transducer (GE Vingmed, Horten, Norway) by standard methods according to the guidelines of the American Society of Echocardiography and the European Association of Cardiovascular Imaging [10]. AV annulus diameter was measured in the parasternal long-axis view on the mid-systole frame. Left ventricular (LV) end-diastolic diameter and end-systolic diameters were measured from a parasternal long-axis view. LV ejection fraction (LVEF) was measured by the modified Simpson method in the apical view. The left atrial volume index (LAVI) was measured by the area-length method in the apical view. AV peak pressure gradient (PG) was measured from the trans-aortic velocity flow curve using continuous wave Doppler. Postoperative AV EOA was calculated with the continuity equation using LV stroke volume (SV) and AV velocity time integral (VTI). SV was calculated by the pulse wave Doppler method as LVOT area × LVOT VTI.

### 2.2. Transesophageal Echocardiography

**Figure 1:**
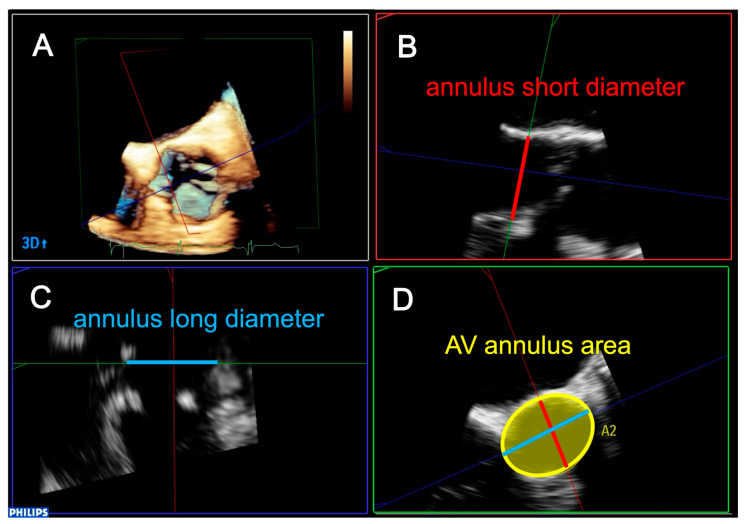
Methods of three-dimensional analysis of aortic valve using transesophageal echocardiography. From preoperative three-dimensional transesophageal echocardiographic (3D TEE) data, aortic annulus area was measured using 3DQ software at mid-diastolic phase (**A**). Two-dimensional (2D) long-axis view: aortic annulus short diameter (red line) (**B**) and long diameter view (blue line) (**C**). 2D short-axis view (**D**), AV annulus area (yellow circle). AV; aortic valve.

TEE was performed using the EPIQ system with an X7-2t or X8-2t transducer or the iE33 system with an X7-2t transducer (Philips Medical Systems, Andover, MA, USA). We obtained 3D full-volume AV data during breath-holding for six heartbeat compositions or four heartbeats in atrial fibrillation patients. The view was optimized for depth and gain setting before 3D acquisition for high spatial and temporal resolution data. For preoperative 3D data, we performed annulus analysis using 3DQ software version 15.0 (Philips Medical Systems, Andover, MA, USA). Two orthogonal long-axis planes of the AV were extracted from 3D datasets using multiplanar reconstruction mode (Figure 1A). The third plane perpendicular to both of the long-axis planes was manipulated to obtain the orthogonal 2D short-axis cutting plane of the AV annulus (Figure 1B,C). After choosing the mid-systole frame, we obtained the 2D transverse plane at the level of the aortic annulus, which is defined as the plane which includes all of lowest cusp hinge points. We then traced the aortic annulus and consequently measured the preoperative AV annulus area (Figure 1D).

**Figure 2:**
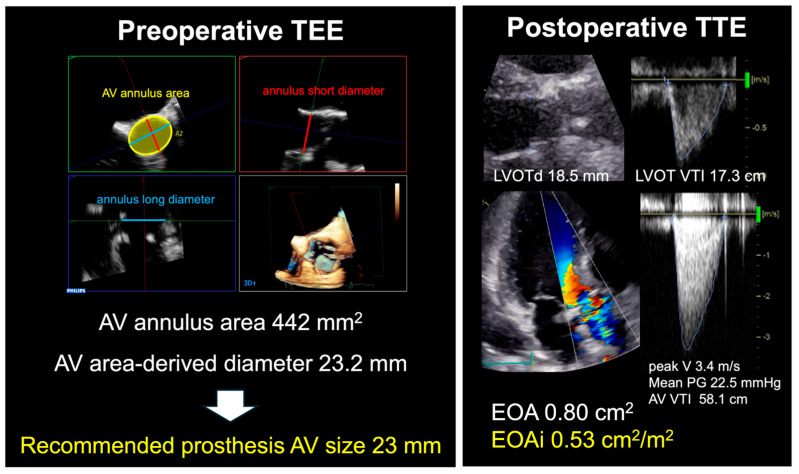
Representative case of severe postoperative prosthesis-patient mismatch (PPM). The patient was a 68-year-old female with aortic stenosis. The aortic valve (AV) annulus area was 442 mm^2^ (yellow circle) and the AV annulus area-derived diameter was calculated as 23.2 mm. This patient was implanted with a 21-mm prosthetic valve, which was smaller than the recommended prosthesis AV size. AV annulus diameter was reduced by 9.5% after the procedure and severe PPM occurred. TEE; transesophageal echocardiography, AV; aortic valve, TTE; transthoracic echocardiography, V; velocity, PG; pressure gradient, EOAi; effective orifice area index.

The AV annulus area-derived diameter was calculated using the equation (2 × [AV area/π]1/2) and used as the preoperative AV annulus diameter. Postoperative AV annulus diameter was defined using the prosthetic valve size. The AV annulus diameter change ratio between pre- and post-SAVR (%) was calculated using the equation (100 − [post-AV annulus diameter/pre-AV annulus diameter] × 100). The recommended prosthesis AV size was defined as the largest size below the AV annulus area-derived diameter by preoperative 3D TEE data (Table 1). A representative case was presented in Figure 2.

### 2.3. Operative Procedure

SAVR in this study was performed via a median sternotomy approach (*n* = 191, 93%) or a minimally invasive cardiac surgery approach (*n* = 14, 7%). The type of valve was chosen based on the surgeon’s preference according to AV morphology. Valve size was selected based on measurement with the provided sizer and also based on the product-specific correspondence table, which was expected to provide an EOAi of 0.85 or higher.

### 2.4. Statistical Analysis

Continuous variables are expressed as mean ± standard deviation and categorical variables are summarized as percentages and counts. Continuous variables between patient groups were compared using the Student *t*-test. Differences in proportions were examined by Fisher’s exact test. A *p*-value less than 0.05 was considered significant. The correlation between EOAi and AV annulus diameter change ratio between pre- and post-SAVR was assessed using Spearman’s correlation coefficient. The associated variables in univariate analyses (*p* < 0.05) were included in the multivariable logistic regression analysis model to identify predictors of severe PPM after SAVR. All statistical analyses were performed using the JMP software program, version 18.1.1 (SAS Institute Inc., Cary, NC, USA).

## 3. Results

A total of 6 patients (2.9%) were determined to have severe PPM after SAVR (Figure 2).

Clinical characteristics of patients with and without severe PPM are compared in Table 2. The PPM group was younger than the group without PPM.

### 3.1. Preoperative Echocardiographic Parameters

Preoperative TTE and TEE parameters in patients with and without severe PPM are compared in Table 3. Preoperative LA volume, LV size, LV function, and AV morphological parameters in the two groups were similar. Preoperative AV annulus area-derived diameter was 24.9 ± 2.4 mm in the group with severe PPM and 23.9 ± 2.4 mm in the group without severe PPM.

### 3.2. Types and Size of Implanted Prosthetic Valve

Types and size of implanted prosthetic valve in patients with and without severe PPM are compared in Table 4.

Inspiris RESILIA (Edwards Lifesciences, Irvine, CA, USA) was used for 163 (79.5%) patients; Carpentier-Edwards Perimount (CEP) Magna Ease (Edwards Lifesciences, Irvine, CA, USA) was used for 26 (12.7%) patients; Avalus (Medtronic Inc., Minneapolis, MN, USA) was used for 15 (7.3%) patients; and Crown PRT (Corcym Group, Burnaby, Canada) was used for 1 (0.5%) patient. Comparison of types and label sizes of implanted prosthetic valves in patients with and without severe PPM showed no significant differences between the two groups. Annulus diameter of implanted prosthetic valve was 22.3 ± 2.1 mm in the group with severe PPM and 23.1 ± 2.2 mm in the group without severe PPM. Overall, 98 patients (48%) received the recommended prosthetic valve size, 46 patients (22%) received undersized valves, and 61 patients (30%) received oversized valves. Among the 46 patients with undersized valves, 21 patients had aortic stenosis, 23 patients had aortic regurgitation, and 2 patients had both conditions, preoperatively. The proportions of these conditions were not significantly different from those observed in the overall patient population.

### 3.3. Surgical Procedures

Surgical procedures in patients with and without severe PPM are compared in Table 5.

There was no significant difference between the two groups with regard to suture annular position or concomitant procedure.

### 3.4. Postoperative Echocardiographic Parameters

Parameters on postoperative TTE in patients with and without severe PPM are compared in Table 6.

Postoperative TTE was performed an average of 6.3± 1.8 days after surgery. EOAi calculated from the continuous equation was 0.61 ± 0.04 cm^2^/m^2^ in the group with severe PPM and 1.14 ± 0.30 cm^2^/m^2^ in the group without severe PPM. AV mean PG was 17.9 ± 8.0 mmHg in the group with severe PPM and 9.6 ± 4.1 mmHg in the group without severe PPM. AV annulus diameter change ratio between pre- and post-SAVR was significantly larger in the group with severe PPM than in the group without severe PPM (10.4 ± 3.6% vs. 3.0 ± 5.6%, *p* = 0.002). The ratio of use of a prosthesis valve with a smaller than recommended prosthesis size was higher in the severe PPM group than in the without severe PPM group (83.3% vs. 20.6%, *p* = 0.002).

### 3.5. Correlations Between EOAi and AV Annulus Diameter Parameters

**Figure 3:**
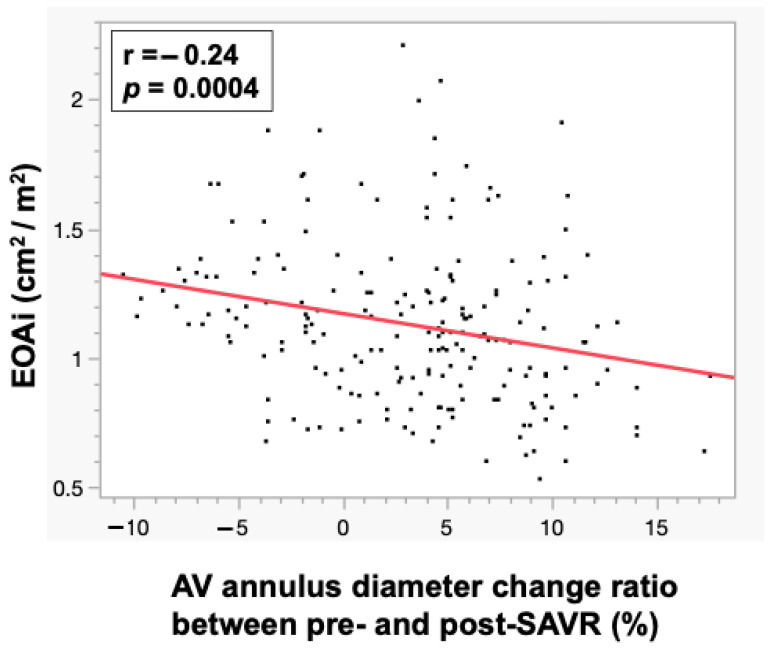
Correlation between effective orifice area index and aortic valve annulus area parameters. Significant correlations were seen between effective orifice area index (EOAi) and aortic valve (AV) annulus diameter change ratio pre- and post-surgical aortic valve replacement (SAVR) (r = −0.24, *p* = 0.0004).

A significant correlation was seen between EOAi and AV annulus diameter change ratio between pre- and post-SAVR (r = −0.24, *p* = 0.0004) (Figure 3).

### 3.6. Logistic Regression Analysis for Prediction of Severe PPM

Univariate and multivariate logistic regression analyses are shown in Table 7.

Univariate analysis identified age, AV annulus diameter change ratio be-tween pre- and post-SAVR and use of a valve with a smaller than recommended prosthesis AV size as significant predictors of PPM. Among these three, AV annulus diameter change ratio and use of a prosthesis smaller than the recommended prosthesis AV size exhibit multicollinearity, we selected use of a prosthesis smaller than the recommended prosthesis AV size, which has greater clinical relevance, for multivariate analysis. Analysis of risk factors for severe PPM using a multivariable logistic regression model, including age and the using prosthesis valve with smaller than recommended prosthesis AV size, showed that only the using prosthesis valve with smaller than recommended prosthesis AV size was an independent predictor of severe PPM (the ratio of using prosthesis valve with smaller than recommended prosthesis AV size increase: odds ratio 19.3, 95% confidence interval 2.14–174.5, *p* = 0.008).

## 4. Discussion

This study examined the utility of AV annulus area-derived diameter and the recommended prosthetic AV size as measured by preoperative 3D TEE analysis in preventing severe PPM after SAVR. Six of 205 patients (2.9%) who underwent SAVR using a bioprosthetic valve showed severe PPM. Although the study had limited statistical power due to the low incidence of PPM, particularly for the logistic regression analysis, it provided an important indicator for PPM prevention. The group with severe PPM showed a greater decrease in AV annulus diameter change ratio between pre- and post-SAVR than the group without severe PPM. Use of a prosthesis valve with a smaller than recommended prosthesis AV size was more frequent in the group with severe PPM than in that without severe PPM. Use of a valve with a smaller than recommended prosthesis AV size was an independent predictor of severe PPM. Our findings suggest that when performing SAVR, use of a prosthetic valve larger than the size recommended by 3D TEE may prevent the occurrence of severe PPM. The inter- and intra-observer variability for 3D-TEE measurements, assessed by intraclass correlation coefficient (ICC), demonstrated excellent reliability, with ICC (2, 2) = 0.94 and ICC (1, 2) = 0.98, based on data from ten randomly selected patients. These findings indicate that the AV diameter measured using 3D-TEE is a reliable and useful parameter.

### 4.1. Incidence of PPM in SAVR

In this study, all patients had prosthetic valves larger than the recommended prosthesis AV size based on body surface area. However, severe PPM was observed in 6 of 205 patients (2.9%), a finding comparable to or even lower than reported in larger studies [2,3,11,12]. This result can be attributed to the use of improved prosthetic valves in this study, which allow for a larger EOA compared to those used in previous studies. Additionally, we speculate that surgeons considered implanting larger valves to facilitate future transcatheter aortic valve-in-surgical aortic valve procedures if reoperation becomes necessary [13].

### 4.2. Risk Factors for PPM After SAVR

Previously reported predictors for PPM include older age, female sex, hypertension, diabetes, renal failure, larger BSA, larger body mass index, and utilization of a bioprosthetic valve [14]. These predictors appear to include the conflicting parameters of older female and larger body size, suggesting the complexity of the clinical picture in these patients.

In this study, the PPM group was significantly younger and without a larger BSA than the non-PPM group. There were several young patients with annuloaortic ectasia in the PPM group who developed PPM because the necessary EOA could not be obtained despite use of the largest size prosthetic valve. Thus, in cases in which PPM is predicted based on the preoperative change in AV annulus diameter, techniques that avoid PPM should be considered, such as AVR with aortic root enlargement or valve-sparing root replacement. In contrast, when PPM after SAVR is predicted in elderly patients, transcatheter aortic valve replacement (TAVR) is a useful option to obtain a larger EOA than SAVR. Preoperative case-by-case consideration of PPM risk using the AV annulus diameter change ratio is considered a useful tool for preventing PPM and improving patient prognosis.

### 4.3. Importance of Preventing PPM in SAVR

PPM following AVR is a known poor prognostic factor after surgery, with increased long-term mortality and a higher risk of hospitalization due to heart failure [12,15]. PPM is associated with poor long-term prognosis for two main reasons: first, its occurrence prevents adequate afterload reduction, leading to decreased coronary flow reserve and subsequent left ventricular myocardial impairment [16]; and second, the increased transvalvular flow velocity caused by PPM is thought to contribute to early structural valve deterioration (SVD) [17]. PPM leads to a decreased incidence of LVEF, significant LV hypertrophy, and paradoxical low-flow gradient, and serves as a more pronounced poor prognostic factor in patients younger than 70 years [18]. Preventing severe PPM after SAVR is therefore crucial.

This study shows that pre-and postoperative valve annulus diameter change ratio is more strongly associated with severe PPM than postoperative AV annulus diameter. In addition, the postoperative AV annulus diameter did not differ between cases with and without severe PPM, which showed that the effect of placing a smaller AV annulus diameter relative to the preoperative diameter was most strongly associated with severe PPM.

Use of prosthetic valves smaller than the recommended prosthetic AV size as calculated from the preoperative valve annulus area was most strongly associated with severe PPM. Accordingly, selection of a prosthetic valve equal to or larger than the recommended prosthetic AV size appears effective in preventing severe PPM. For patients with a narrow annulus for whom a prosthetic valve of the recommended size cannot be used, aortic root enlargement can be planned preoperatively to avoid PPM [19].

### 4.4. Usefulness of the Recommended Prosthetic AV Size in SAVR

A previous study reported the presence of a good correlation between the preoperative AV annulus size and implanted prosthetic valve size in TAVR, but no such correlation in SAVR cases [6]. This discrepancy arose because TAVR relied on preoperative imaging to determine the appropriate prosthetic valve size intraoperatively [20], whereas SAVR is often dependent on the intraoperative judgment of the surgeon. In univariate analysis of this study, neither postoperative AV annulus diameter nor post operative AV annulus diameter/BSA was identified as a predictor of PPM. This suggests that the relative change in valve dimensions before and after intervention may be more relevant than the absolute postoperative annular size itself. Therefore, calculating the optimal prosthetic valve size based on the preoperative AV annulus size might also benefit SAVR cases. Our present study demonstrated a significant correlation between postoperative EOAi and AV annulus area diameter ratio pre- and post-SAVR. This suggests that preoperative evaluation of the recommended prosthetic AV size calculated on the AV annulus area in TEE may aid selection of an appropriate prosthetic AV size which avoids postoperative PPM. Determining the prosthetic AV size based on this case-specific value would allow for theory-based SAVR, as follows:

1. From our study, it was revealed that use of a valve with a smaller than recommended prosthesis AV size influences postoperative EOAi and may be an independent predictive factor for severe PPM.
2. Although the choice of prosthetic valve size is currently determined by the surgeon using a sizer during surgery, size should be preoperatively determined from preoperative imaging in future.

On the other hand, AV annulus diameter change ratio and EOAi demonstrated only a weak correlation. This is presumably because factors other than the valve ring size—such as the type of prosthetic valve and suture site—also influence the calculation of EOAi. Because the number of cases in this study was limited, we were unable to perform a comprehensive analysis of these factors, which represents an area for future investigation.

### 4.5. Application to Mechanical Valves and TAVR

Mechanical valves were excluded from this study because they are designed to have a larger EOA compared with bioprosthetic valves [21]. Although the incidence of PPM is lower with mechanical valves than with bioprosthetic valves, PPM can still occur [21]. Therefore, the findings of this study may be applicable to preventing PPM in patients receiving mechanical valves too.

In TAVR, prosthetic valve sizing is typically determined preoperatively using contrast-enhanced computed tomography (CT) [22]. However, in patients for whom contrast CT is contraindicated, 3D TEE, as utilized in this study, may serve as an alternative imaging modality for valve sizing.

### 4.6. Limitations

This study has several limitations. First, the study was conducted under a retrospective design with a relatively small number of patients, and the power of all statistical analyses was insufficient. Especially, the power of multivariate analysis was limited. Second, SAVR in this study was conducted by several surgeons, which likely resulted in differences in technique in aspects other than prosthetic valve selection. Finally, various types of prosthetic valves were employed in this study, indicating the presence of structural heterogeneity among the valves.

## 5. Conclusions

The recommended prosthetic AV size calculated on 3D TEE is useful in determining the optimal prosthetic valve size to prevent PPM.

## Figures and Tables

**Table 1:** Definition of recommended prosthetic aortic valve size.

3D TEE-Derived Annulus Diameter (mm)	Recommended Prosthesis AV Size (mm)
≤20.9	19
21.0–22.9	21
23.0–24.9	23
25.0–26.9	25
27.0–28.9	27
29.0≤	29

3D TEE, three-dimensional transesophageal echocardiography; AV, aortic valve.

**Table 2:** Comparison of patient characteristics between patients with and without severe PPM.

	Severe PPM Group (*n* = 6)	Without Severe PPM Group (*n* = 199)	*p* Value
Age, years	66 ± 7	73 ± 7	**0.040**
Men, *n* (%)	4 (67)	125 (63)	1.000
Body weight, Kg	56 ± 14	57 ± 12	0.828
Body height, cm	161 ± 10	161 ± 10	0.932
Body surface area, m^2^	1.64 ± 0.22	1.58 ± 0.19	0.456
Hypertension, *n* (%)	4 (67)	151 (76)	0.635
Diabetes mellitus, *n* (%)	1 (2)	36 (18)	1.000
Chronic kidney disease, *n* (%)	4 (67)	102 (51)	0.684
Atrial fibrillation, *n* (%)	2 (33)	59 (30)	1.000
Coronary artery disease, *n* (%)	2 (33)	51 (26)	0.650

Data are expressed as mean ± SD or *n* (%). Bold values indicated statistical significance (*p*-value < 0.05). PPM, prosthesis-patient mismatch.

**Table 3:** Comparison of parameters of preoperative transthoracic echocardiography and transesophageal echocardiography between patients with and without severe PPM.

	Severe PPM Group (*n* = 6)	Without Severe PPM Group (*n* = 199)	*p* Value
Transthoracic echocardiography			
LAVI (mL/m^2^)	55.2 ± 30.4	47.9 ± 35.7	0.620
LVEDD (mm)	58.2 ± 12.0	53.6 ± 10.3	0.281
LVESD (mm)	42.5 ± 10.0	38.8 ± 11.0	0.415
LVEF (%)	43.9 ± 18.5	54.8 ± 13.5	0.054
AV annulus diameter (mm)	21.5 ± 2.4	22.2 ± 2.6	0.502
Valvular disease types			0.783
Aortic stenosis	3 (50.0)	75 (37.7)	
Aortic regurgitation	3 (50.0)	112 (56.3)	
Aortic stenosis and regurgitation	0 (0)	11 (5.5)	
Transesophageal echocardiography			
AV annulus area (mm^2^)	492 ± 93	451 ± 89	0.265
AV annulus area-derived diameter (mm)	24.9 ± 2.4	23.9 ± 2.4	0.272
Recommended prosthesis AV size (mm)	24.3 ± 2.1	22.9 ± 2.4	0.141

LAVI, left atrial volume index; LVEDD, left ventricular end-diastolic diameter; LVESD, left ventricular end-systolic diameter; LVEF, left ventricular ejection fraction; AV, aortic valve; PPM, prosthesis-patient mismatch.

**Table 4:** Comparison of implanted valve types between patients with and without severe PPM.

	Severe PPM Group (*n* = 6)	Without Severe PPM Group (*n* = 199)	*p* Value
Valve type			0.251
Inspris RESILIA	4 (66.7)	159 (79.9)	
CEP Magna Ease	2 (33.3)	24 (12.1)	
Avalus	0 (0)	15 (7.5)	
Crown	0 (0)	1 (0.5)	
Valve size			
19	1 (17.7)	20 (10.1)	0.843
21	1 (17.7)	42 (21.1)	
23	3 (50.0)	64 (32.2)	
25	1 (17.7)	56 (28.1)	
27	0 (0)	17 (8.5)	
Mean valve size (mm)	22.3 ± 2.1	23.1 ± 2.2	0.419

PPM, prosthesis-patient mismatch.

**Table 5:** Comparison of surgical procedures between patients with and without severe PPM.

	Severe PPM Group (*n* = 6)	Without Severe PPM Group (*n* = 199)	*p* Value
Minimally invasive approach	0 (0)	14 (7.0)	1.000
Suture annular position			0.703
Supra	3 (50.0)	78 (39.2)	
Intra	3 (50.0)	119 (59.8)	
Para	0 (0)	2 (1.0)	
Bentall procedure	0 (0)	21 (10.6)	1.000
Concomitant procedure			
Mitral valve surgery	1 (16.7)	47 (23.6)	1.000
Tricuspid valve surgery	1 (16.7)	16 (8.0)	1.000

PPM, prosthesis-patient mismatch.

**Table 6:** Comparison of parameters of postoperative transthoracic echocardiography between patients with and without severe PPM.

	Severe PPM Group (*n* = 6)	Without Severe PPM Group (*n* = 199)	*p* Value
**Transthoracic echocardiography**			
LAVI (mL/m^2^)	37.7 ± 7.4	36.7 ± 19.7	0.905
LVEDD (mm)	51.7 ± 8.0	48.2 ± 8.9	0.349
LVESD (mm)	37.8 ± 6.5	35.6 ± 10.1	0.597
LVEF (%)	49.6 ± 14.2	51.0 ± 14.2	0.816
EOAI (cm^2^/m^2^)	0.61 ± 0.04	1.14 ± 0.30	**<0.001**
Peak velocity (m/s)	2.9 ± 0.7	2.1 ± 0.4	**<0.001**
Mean PG (mmHg)	17.9 ± 8.0	9.6 ± 4.1	**<0.001**
**Transesophageal echocardiography**			
AV annulus diameter change ratio between pre- and post-SAVR (%)	10.4 ± 3.6	3.0 ± 5.6	**0.002**
Use of a valve with a smaller than recommended prosthesis AV size (%)	5 (83.3)	41 (20.6)	**<0.001**

Bold values indicated statistical significance (*p*-value < 0.05). LAVI, left atrial volume index; LVEDD, left ventricular end-diastolic diameter; LVESD, left ventricular end-systolic diameter; LVEF, left ventricular ejection fraction; EOAI, effective orifice area index; AV, aortic valve; PG, pressure gradient; AVR, surgical aortic valve replacement; PPM, prosthesis-patient mismatch.

**Table 7:** Univariate and multivariate logistic regression analysis for prediction of severe PPM.

Variable	Univariate		Multivariable	
	OR (95% CI)	*p* Value	OR (95% CI)	*p* Value
Age	0.91 (0.83–0.99)	**0.048**	0.91 (0.82–1.01)	0.071
Male sex	0.78 (0.39–1.56)	0.477		
Body surface area	1.18 (0.77–1.82)	0.459		
Postoperative AV annulus diameter	0.86 (0.59–1.24)	0.418		
Post operative AV annulus diameter/BSA	0.63 (0.34–1.09)	0.105		
AV annulus diameter change ratio between pre- and post-SAVR	1.36 (1.13–1.72)	**<0.001**		
Use of a valve with a smaller than recommended prosthesis AV size	19.27 (2.19–169)	**0.008**	19.3 (2.14–175)	**0.008**

Bold values indicated statistical significance (*p*-value < 0.05). AV, aortic valve; SAVR, surgical aortic valve replacement; BSA, body surface area; OR, odds ratio; CI, confidence interval.

## Data Availability

No data will be shared related to this study.
